# Different Non-Structural Carbohydrates/Crude Proteins (NCS/CP) Ratios in Diet Shape the Gastrointestinal Microbiota of Water Buffalo

**DOI:** 10.3390/vetsci8060096

**Published:** 2021-05-31

**Authors:** Rubina Paradiso, Giorgia Borriello, Sergio Bolletti Censi, Angela Salzano, Roberta Cimmino, Giorgio Galiero, Giovanna Fusco, Esterina De Carlo, Giuseppe Campanile

**Affiliations:** 1 Department of Animal Health, Istituto Zooprofilattico Sperimentale del Mezzogiorno, 80055 Portici, Italy; rubina.paradiso@izsmportici.it (R.P.); giorgia.borriello@izsmportici.it (G.B.); giorgio.galiero@cert.izsmportici.it (G.G.); giovanna.fusco@cert.izsmportici.it (G.F.); esterina.decarlo@cert.izsmportici.it (E.D.C.); 2 Cosvitec Scarl, 80100 Naples, Italy; sergiobolletti@cosvitec.eu; 3 Department of Veterinary Medicine, University of Naples “Federico II”, 80137 Naples, Italy; giucampa@unina.it; 4 Italian Buffalo Breeders Association, 81100 Caserta, Italy; r.cimmino@anasb.it

**Keywords:** gastrointestinal microbiota, rumen, large intestine, feces, water buffalo, fiber, diet, food industry by-products, tomato peel

## Abstract

The microbiota of the gastrointestinal tract (GIT) are crucial for host health and production efficiency in ruminants. Its microbial composition can be influenced by several endogenous and exogenous factors. In the beef and dairy industry, the possibility to manipulate gut microbiota by diet and management can have important health and economic implications. The aims of this study were to characterize the different GIT site microbiota in water buffalo and evaluate the influence of diet on GIT microbiota in this animal species. We characterized and compared the microbiota of the rumen, large intestine and feces of water buffaloes fed two different diets with different non-structural carbohydrates/crude proteins (NSC/CP) ratios. Our results indicated that Bacteroidetes, Firmicutes and Proteobacteria were the most abundant phyla in all the GIT sites, with significant differences in microbiota composition between body sites both within and between groups. This result was particularly evident in the large intestine, where beta diversity analysis displayed clear clustering of samples depending on the diet. Moreover, we found a difference in diet digestibility linked to microbiota modification at the GIT level conditioned by NSC/CP levels. Diet strongly influences GIT microbiota and can therefore modulate specific GIT microorganisms able to affect the health status and performance efficiency of adult animals.

## 1. Introduction

In ruminants, the microbiota of the gastrointestinal tract (GIT) play a crucial role in host health and production efficiency [1,2,3]. The GIT, particularly the rumen, harbors a rich and diverse microbial community, which lives in a symbiotic relationship with the host. In this interaction, bacterial enzymes perform tasks not provided by the host genome, thus expanding the spectrum of metabolic functions and capabilities of the host. The GIT microbiota are responsible for the degradation of proteins, lipids, starch, cellulose, lignin, and hemicellulose [1]. Through polysaccharide digestion, microbes of the GIT produce volatile fatty acids (VFAs), particularly short-chain fatty acids (SCFAs), such as acetate, butyrate, and propionate which act as the main energy intake for the host [4]. Moreover, GIT microbiota strongly influence the development and maturation of the host immune system and can modulate both innate and adaptive immune responses [2].

The microbial community of GIT in ruminants is composed mostly of bacteria but also includes archaea, fungi, protozoa and viruses [5]. Its composition can be influenced by several factors, such as diet [6], animal age, use of antibiotics, host health and welfare, geographic location, zootechnical stress level (housing strategy), and feeding regimen [7,8]. Indeed, microbiota structure has also been shown to be influenced by feed intake levels [9] and frequency of feeding [10], thus suggesting the possibility to manipulate its composition by diet and management. In beef and dairy industries, differences in microbiota structure have been associated with different milking performances [11,12,13], weight gain in calves [14] and methane emissions [15,16]. Microbiota manipulation through diet is, therefore, an interesting area of research, particularly in the agri-food industry, where its application can have important animal health and economic implications. Ruminant-based food production systems currently need to respond to increased human food requests and, on the other hand, to reduce environmental pollution. Animal farming indeed determines food-feed competition, and particularly ruminants are criticized for the lower feed conversion efficiency if compared with monogastric livestock. From this perspective, the possibility of using food industry by-products for ruminant feeding appears to be a promising tool to improve feed- and food-production systems sustainability. Appropriate feed choices can significantly impact ruminant productions as well as methane emissions, therefore alternative feeds must be evaluated for their feed value, animal responses and costs. Numerous food industry by-products are already used for animal feeding, including hulls and those derived from the milling, distillery and brewery industries [17]. Vegetable and fruit residues can be preserved by sun-drying or ensiling in order to be added to the ruminant feed. Through solid-state fermentation, vegetable wastes can acquire more proteins and nutrients, improve feed quality and enhance ensilability, even though constraints in their use can be caused by their moisture content and presence of anti-nutritional factors such as pesticides, mycotoxins, heavy metals and dioxins [18]. Successful results related to the use of ensiled tomato and olive by-products for dairy goats feeding have been previously reported [19]. Southern Italy is a great tomato producer, and tomato pomace (TP), composed of the mixture of peel, core and seeds derived from the tomato industry has been shown to be suitable to completely replace concentrate mixture in male buffaloes, without affecting dry matter intake (DMI), digestibility of nutrients, urinary purine derivatives, microbial protein synthesis and VFAs production in the rumen. Moreover, studies on ensiled TP in ruminants diet demonstrated its ability to reduce methane emissions [19].

The advent of high throughput sequencing technologies has now made it possible to investigate the bacterial species present in different matrices through a culture-independent method, thus allowing the identification and classification of all the present species, including unculturable microbes [20]. This approach proved to be extremely suitable for the study of GIT, particularly rumen microbiota, since it has been estimated that only 20% of this complex microbial community can be cultured by standard techniques [21]. The studies based on the 16S rRNA gene sequencing allow the identification of low abundance species enabling the analysis of rare microbial communities. Therefore this technique makes it possible to characterize shifts in complex microbial communities in response to external factors, such as diet.

Therefore, the aims of this study were (i) to characterize the different GIT site microbiota in water buffaloes, and (ii) to evaluate the influence of diet on GIT microbiota in water buffaloes during their dry period characterized by non-structural carbohydrate/crude protein (NSC/CP) of different ratios obtained with the addition of ensiled tomato peel.

## 2. Materials and Methods

### 2.1. Animals

The animals enrolled in the trial were 20 Italian Mediterranean female water buffaloes (*Bubalus bubalis*) in their dry period, with an average age of 7.1 ± 1.2 years, a number of calvings of 3.7 ± 1.3 and for which slaughtering was already scheduled. Animals were maintained in pens with a concrete floor, divided into two homogenous groups and fed diets with different non-structural carbohydrates/crude proteins (NSC/CP) ratios (Table 1). The first group (traditionally fed; *n* = 10) received a standard diet with an NSC/CP ratio of 1.9. The second group (alternatively fed; *n* = 10) received a novel diet with an NSC/CP ratio of 2.3. Individual feedstuff and refusals were sampled once per week and analyzed according to AOAC [22]. For both groups, the different diets were administered for 7 weeks, including a 3-week adaptation phase, followed by a 4-week experimental period. All the procedures performed followed the common clinical practices [23] and received institutional approval from the Ethical Animal Care and Use Committee of University of Naples “Federico II” (Protocol No. 996072017); moreover, the farmer was previously informed and in agreement with the purposes and methods used.

### 2.2. Diet Digestibility

The organic matter (OM) and cell wall digestibility (CWD) were evaluated every week by using acid insoluble ashes as an internal marker [24]. Fecal samples (200 g) were collected daily for five consecutive days for each animal. In order to avoid sample fermentation, immediately after collection, feces samples were frozen at −20 °C and stored until further processing. Before the analysis, for each animal, individual feces samples were pooled and processed for OM and CWD determination as elsewhere described [22].

### 2.3. Sample Collection and DNA Extraction

At the end of the experimental period animals, which were at the end of their reproductive and productive careers, were slaughtered and samples of the rumen, large intestine and feces were collected for molecular studies. Each sample was labeled and stored at −80 °C until analysis. DNA was extracted by the DNeasyPowerSoil Kit (MO BIO Laboratories, Inc, Carlsbad, CA, USA) according to the manufacturer’s instructions, also including negative extraction controls. DNA was quantified using a high-sensitivity Qubit™ fluorometer. 

### 2.4. Amplification and Sequencing

The 16S rRNA gene was amplified by the 16S Ion Metagenomics kit (Life Technologies, Carlsbad, CA, USA) following the manufacturer’s instructions. Briefly, two separate PCR reactions were carried out, amplifying, respectively, the V2-4-8 and V3-6, V7-9 regions. Each PCR reaction included 5 ng of microbial DNA and was amplified by a thermal profile consisting of the following steps: 1 cycle at 95 °C for 10 min, 30 cycles consisting of 95 °C for 30 s, 58 °C for 30 s, 72 °C for 20 s, and a final extension cycle at 72 °C for 7 min. After amplification, PCR products were purified using the Agencourt AM Pure beads (Beckman Coulter Inc, Atlanta, GA, USA), eluted in Low TE buffer and quantified by the Qubit dsDNA HS Assay kit (Life Technologies, Carlsbad, CA, USA). Reactions included DNA extraction and PCR amplification of negative controls to evaluate the effect of possible contamination along the processing workflow [25,26,27]. For library preparation, for each sample, 50 ng of DNA from each PCR reaction were pooled to have 100 ng of total DNA to be used for further processing. Libraries were barcoded using Ion Xpress Barcodes Adapters (Life Technologies) and amplified in an emulsion PCR on the One-Touch 2 and One-Touch ES systems (Life Technologies) according to the manufacturer’s instructions. Sequencing was performed on the Ion Personal Genome Machine (PGM) using the Ion 318 Chip kit V2 (Life Technologies). 

### 2.5. Data Analysis

After sequencing, reads pre-processing for quality control was performed by DADA2 (DADA2 denoise-pyro plugin) to denoise, remove primers, de-replicate single-end sequences, remove chimeras and exclude low quality reads [28,29]. Based on the quality control check, filtered and de-noised reads were resolved to high-resolution Amplicon Sequence Variants (ASVs), which represent, as closely as possible, the original biological sequence of the sequenced amplicon [28]. The downstream taxonomic analysis at phylum, family and genus level was carried out by the QIIME 2-2020.6 software. For this purpose, multiple sequence alignment of representative ASVs sequences was carried out using the QIIME 2 cluster-features-closed-reference tool (qiime v search cluster-features-closed-reference plugin) [30,31,32]. ASVs were taxonomically classified using a Naïve Bayes classifier, pre-trained on SILVA 138 reference sequences clustered at 99% similarity (QIIME2 feature-classifier classify-sklearn plugin) [33,34]. FastTree [35] software was then used to infer unrooted and subsequently rooted maximum-likelihood phylogenetic trees representing the phylogenetic relatedness of ASVs (QIIME2 phylogeny align-to-tree-mafft-fasttree plugin). Chloroplasts and not classified sequences were excluded from the analysis, given that the total number of found sequences was negligible. After reads pre-processing of the 60 samples included in the study (20 rumen content, 20 large intestine content and 20 feces), the total number of sequences was 15,338,707, with a mean number of sequences of 248,294 per sample in rumen content (median number: 228,192 sequences, minimum number: 123,226 and maximum number: 471,806), 238,019 mean number in large intestine content (median number: 191,351, minimum number: 72,802 and maximum number: 421,725), and 280,621 mean number in feces (median number: 224,171 sequences, minimum number: 122,034 and maximum number: 829,777). Negative controls were sequenced in order to evaluate the presence of eventual contamination [27,28,29]. All the features found in negative controls (Supplementary Table S1) were either absent or present in a negligible number of analyzed samples. Based on this evidence and the best practices for analyzing microbiomes [27,28,29], and in consideration of similar studies in the literature [36,37], we decided to filter out low abundance ASVs by removing all the features with a minimum frequency <150. Following taxonomic analysis, the analysis of the composition of microbiomes (ANCOM) implemented through the QIIME2 ANCOM plugin was carried out to evaluate the presence of statistical differences of population abundance between groups. ANCOM calculates pairwise log-ratios between combinations of taxa and considers how many times (W) the null hypothesis (no difference between each pairwise comparison of taxa) is violated [38]. The ANCOM algorithm determines significance by plotting calculated F-statistics on the x-axis and W-statistics on the y-axis. The F-statistics is a measure of the effect size difference for a particular species between the study groups (diet in our case), and the W-statistic is the strength of the ANCOM test for the tested number of species, with W value representing the number of times that the null hypothesis (no difference between groups) is rejected. To perform this analysis the taxonomic features occurring in fewer than 10 samples or with frequencies below 50 were removed in the input step. Identification of specific features of interest was performed by BLAST analysis [39]. Alpha diversity analysis between body sites, based on observed ASVs (a measure of ASVs abundance) and Pielou evenness index (a quantitative measure of relative evenness of species richness) was performed using the Mann–Whitney test for unpaired samples. Samples from different body sites were also analyzed for beta diversity Principal Coordinates Analyses (PCoA) using Bray–Curtis (a quantitative measure of community dissimilarity), unweighted (a qualitative measure of community dissimilarity incorporating phylogenetic relationships between the features) and weighted (a quantitative measure of community dissimilarity incorporating phylogenetic relationships between the features) UniFrac distances matrices in the QIIME 2 software [40]. Group’s dissimilarity was tested both by Permutational Multivariate Analysis of Variance (PERMANOVA) and Permutational Analysis of Multivariate Dispersions (PERMDISP). PERMANOVA is a non-parametric multivariate statistical test used to assess whether the centroids and dispersion of the groups are equivalent for all groups. The null hypothesis of this test is that the metric centroid does not differ between groups. PERMDISP is a multivariate test that evaluates the homogeneity of dispersion within groups [41]. The null hypothesis of this test is that the average within-group dispersion is the same in all groups. 

Data on digestibility of crude protein, fat content, ash, neutral detergent fiber and organic matter were analyzed by multivariate analysis of variance, general linear model. Diet (group) was used as the main factor. Groups were tested within and between sampling. Results are expressed as mean ± standard error (SE). A statistically significant difference was accepted at *p* < 0.05. 

## 3. Results

### 3.1. Gastrointestinal Microbiota in Traditionally Fed Water Buffaloes

**Figure 1:**
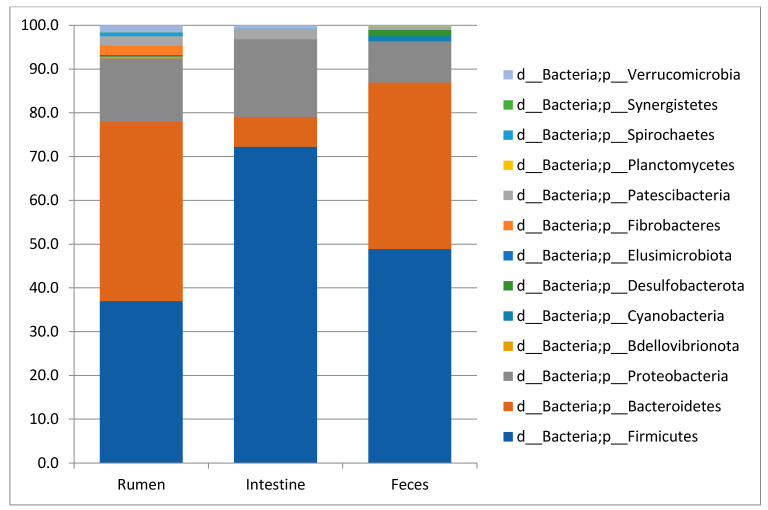
Taxonomy plots at Phylum level of the rumen, large intestine and feces from traditionally fed buffaloes. Relative abundance (mean relative frequency) of the bacterial Phyla identified in different GIT (rumen, large intestine and feces) from traditionally fed water buffaloes (*n* = 10). In the taxa list reported on the right of the figure, the letter d_ indicates Domain, p_ indicates Phylum.

The taxonomic analysis of 10 rumen content, 10 large intestine content and 10 feces samples from traditionally fed water buffaloes exhibited the presence of 13, 11 and 9 phyla, respectively. In all sample types, the most abundant (with mean relative frequencies > 2%) phyla were Bacteroidetes, Firmicutes and Proteobacteria in variable order depending on the specific body site (Figure 1). In the rumen, Bacteroidetes displayed a mean frequency value of 40.9 ± 5%, Firmicutes 37 ± 5.6%, Proteobacteria 14.5 ± 5.7%, followed by Fibro bacteres (2.2 ± 1.6%) and Patesci bacteria (2.1 ± 0.7%). In the large intestine, these values were 72.2 ± 19.1% for Firmicutes, 17.6 ± 19.5% for Proteobacteria and 6.9 ± 8.1% for Bacteroidetes, followed by Patesci bacteria with a mean frequency value of 2.4 ± 1.9%, while in feces they were 48.9 ± 5.1% for Firmicutes, 38 ± 5.6% for Bacteroidetes and 9.4 ± 5.6% for Proteobacteria. At the family level, a total of 61 families were observed in the rumen, 59 in the large intestine and 60 in feces. The list of the most abundant families (with mean frequency values >2%) in the different body sites is reported in Table 2. Finally, at the Genus level, a total of 117, 116 and 126 genera were observed with mean frequency values >2% in the rumen, large intestine and feces, respectively, with the most abundant reported in Table 2. 

**Figure 2:**
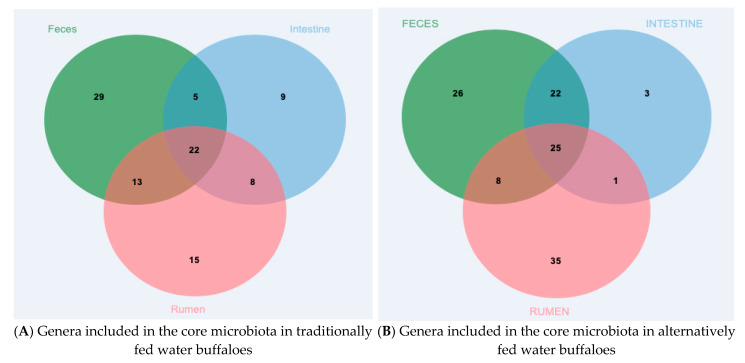
Core microbiota at the Genus level of the rumen, large intestine and feces from differentially fed water buffaloes. Genera shared by at least 80% of samples included in each body site: (**A**) traditionally fed water buffaloes (*n* = 10); (**B**) alternatively fed water buffaloes (*n* = 10).

At the Family level, the core microbiota (intended as the number of taxa shared by at least 80% of samples included in the group) of the three body sites investigated were characterized by 43 families in rumen content, 27 in large intestine content and 46 in feces (Supplementary Figure S1 core Family). Among these families, 21 were shared by all three districts. At the Genus level, the core microbiota included 58 genera in rumen content, 44 in large intestine content and 69 in feces (Figure 2A), with 22 genera shared by all three body sites. The families and genera included in the core microbiota are listed in Supplementary Table S2 core microbiota.

**Figure 3:**
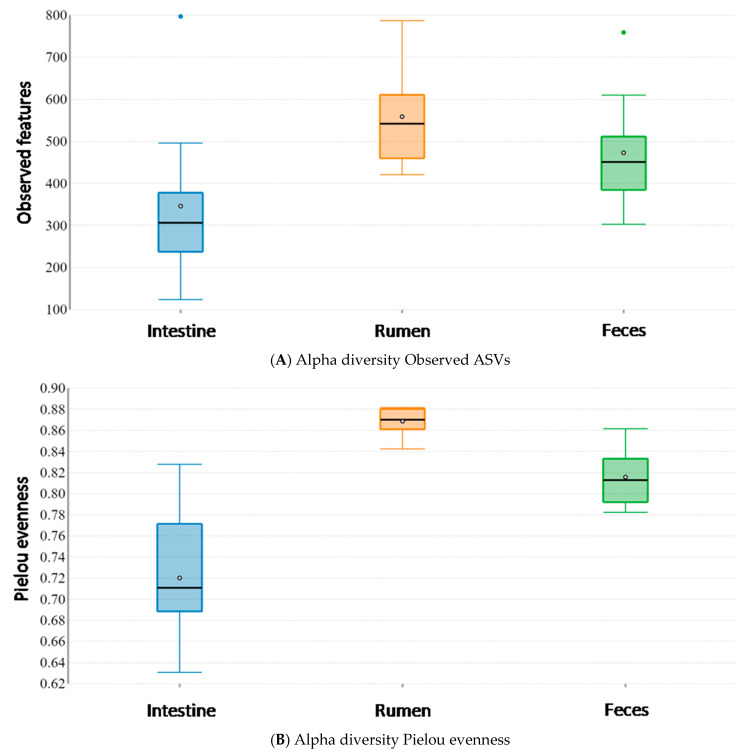
Alpha diversity analysis among the rumen, large intestine and feces from traditionally fed water buffaloes (*n* = 10). Differences in alpha diversity metrics among different body sites: (**A**) Observed ASVs; (**B**) Pielou evenness index.

Alpha diversity analysis of the microbiota in the three different body sites revealed the presence of significant differences among sites, both for the number of Observed ASVs (Kruskal–Wallis pairwise test; Observed ASVs; feces vs. intestine: H = 4.806, *p*-value = 0.028; feces vs. rumen: H = 2.520, *p*-value = 0.112; intestine vs. rumen: H = 7.406, *p*-value = 0.006) (Figure 3A) and microbial evenness (Kruskal–Wallis pairwise test; Pielou evenness index; feces vs. intestine: H = 8.691, *p*-value = 0.003; feces vs. rumen: H = 11.063, *p*-value < 0.001; intestine vs. rumen: H = 14.286, *p*-value < 0.001) (Figure 3B). In particular, the large intestine exhibited the lowest number of Observed ASVs with a lower uniform distribution, while the rumen appeared to be characterized by the highest number of Observed ASVs with a more even distribution. 

**Figure 4:**
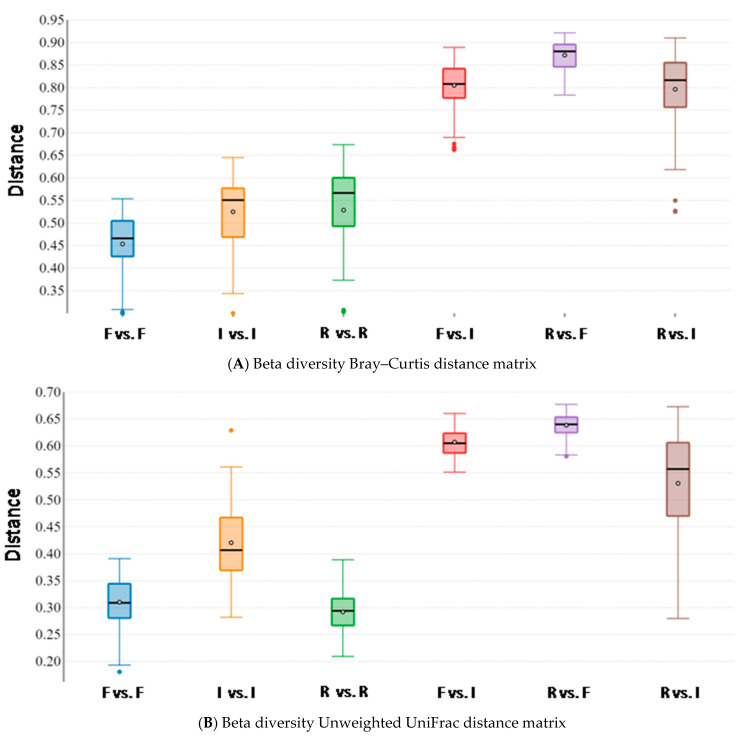

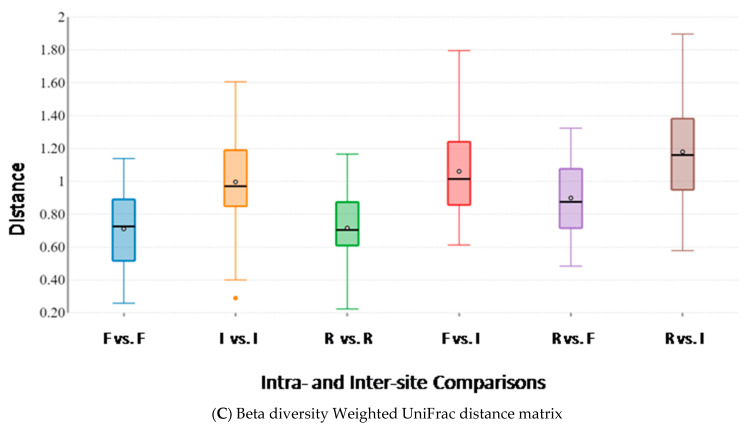
Beta diversity box plots within and between body sites from traditionally fed water buffaloes (*n* = 10). Distances calculated with different matrices: (**A**) Bray–Curtis, (**B**) Unweighted UniFrac and (**C**) Weighted UniFrac. The letter F indicates feces, I indicates large intestine, R indicates rumen.

Beta diversity analysis, performed to assess microbiota similarity within and between body sites, was evaluated by both taxonomic and phylogenetic approaches. Intra- and inter-group distances were compared through measures of species abundance (PERMANOVA; Bray–Curtis; feces vs. intestine: pseudo-F = 17.352; *p*-value = 0.001; feces vs. rumen: pseudo-F = 21.492; *p*-value = 0.001; intestine vs. rumen: pseudo-F = 13.450; *p*-value = 0.001; Figure 4A), taxa phylogeny (PERMANOVA; unweighted UniFrac; feces vs. intestine: pseudo-F = 18.548; *p*-value = 0.001; feces vs. rumen: pseudo-F = 35.166; *p*-value = 0.001; intestine vs. rumen: pseudo-F = 11.696; *p*-value = 0.001; Figure 4B) and both taxa phylogeny and abundance considered together (PERMANOVA; weighted UniFrac; feces vs. intestine: pseudo-F = 5.480; *p*-value = 0.004; feces vs. rumen: pseudo-F = 6.093; *p*-value = 0.001; intestine vs. rumen: pseudo-F = 8.986; *p*-value = 0.001; Figure 4C). Results showed that body sites were characterized by a low degree of dissimilarity within the groups, but exhibited significant differences for all the tested parameters in all the pairwise comparisons between groups, therefore indicating that the structure of the microbiota was highly dissimilar between body sites, for both the taxa present in the microbial communities and their abundances (Figure 4).

### 3.2. Gastrointestinal Microbiota in Alternatively Fed Water Buffaloes 

The analysis of 10 samples of rumen content, large intestine content and feces from alternatively fed water buffaloes exhibited the presence of 13, 8 and 9 phyla, respectively. In particular, in the rumen, the most abundant were Bacteroidetes (42.8 ± 4.7%), Firmicutes (35.3 ± 4.3%) and Proteobacteria (16.4 ± 5.3%), followed by Patescibacteria (2.5 ± 0.7%), while in the large intestine content, the most abundant phyla were Proteobacteria (40.9 ± 28.1%), Firmicutes (36.3 ± 16.2%) and Bacteroidetes (21.6 ± 12.8%), and in feces they were Firmicutes (39.4 ± 9%), Proteobacteria (30.5 ± 14.7%) and Bacteroidetes (28.5 ± 6%). At the Family level, 55, 56 and 60 different families were found in the rumen, large intestine and feces, respectively, with the most abundant (with mean frequency values >2%) reported in Table 2. At the Genus level, 109, 125 and 128 different genera were observed in the rumen, large intestine and feces, respectively, with the most abundant reported in Table 2.

The core microbiota of the three body sites at the Family level consisted of 42 families in the rumen, 33 in the large intestine and 41 in feces; among these families, 22 were shared by at least 80% of samples from each body site (Suppl. Figure S1). At the Genus level, the core microbiota included 69 genera in the rumen, 51 in the large intestine and 81 in feces, with 25 genera shared by at least 80% of samples from each body site (Figure 2B). The families and genera included in the core microbiota are listed in Supplementary Table S2 core microbiota.

### 3.3. Influence of Diet on Water Buffalo Gastrointestinal Microbiota (Traditionally vs. Alternatively Fed Water Buffaloes)

**Figure 5:**
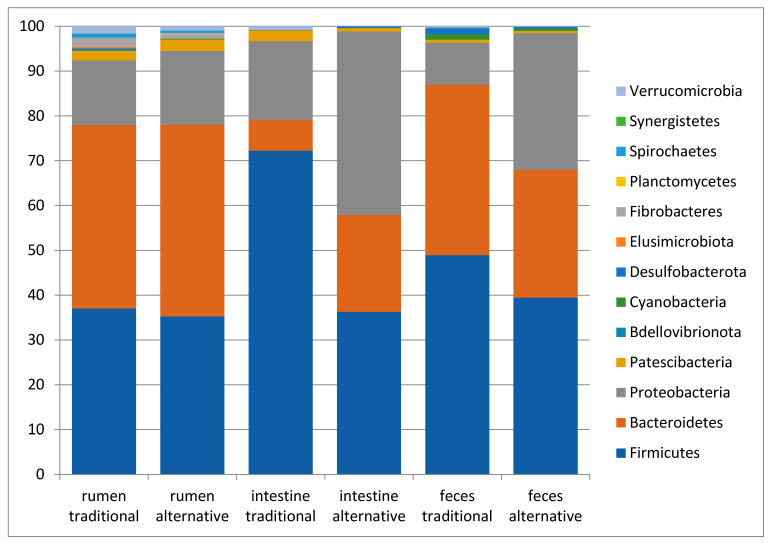
Taxonomy bar plots at Phylum level of the rumen, large intestine and feces from traditionally fed water buffaloes (*n* = 10). Relative abundance (mean relative frequency) of the bacterial Phyla identified in different GIT sites (rumen, large intestine and feces) from traditionally (*n* = 10) and alternatively (*n* = 10) fed water buffaloes.

The comparison of the observed ASV numbers between traditionally and alternatively fed water buffaloes for each body site displayed comparable numbers of observed ASVs for each body site (343.2 ± 69.5 vs. 365.6 ± 108.6 in the rumen, 215.9 ± 112.5 vs. 274.1 ± 147.9 in the large intestine and 325.1 ± 81.2 vs. 327.7 ± 100.2 in feces, respectively, in traditionally and alternatively fed buffaloes). The taxonomic analysis for each body site between traditionally and alternatively fed water buffaloes indicated that, in all samples from both groups, the three most abundant phyla were Bacteroidetes, Firmicutes and Proteobacteria, but with different relative abundances among body sites (Figure 5). In the large intestine and feces, the ratio of Firmicutes and Bacteroidetes (F/B ratio) appeared lower in alternatively fed animals when compared to the traditionally fed ones (Figure 5).

Alpha diversity analysis displayed no significant differences between traditionally and alternatively fed water buffaloes for each body site both in the microbial richness and evenness (Table 3) indicating that the gastrointestinal microbiota in both groups were characterized by comparable numbers of bacterial taxa with an equally even distribution. 

**Figure 6:**
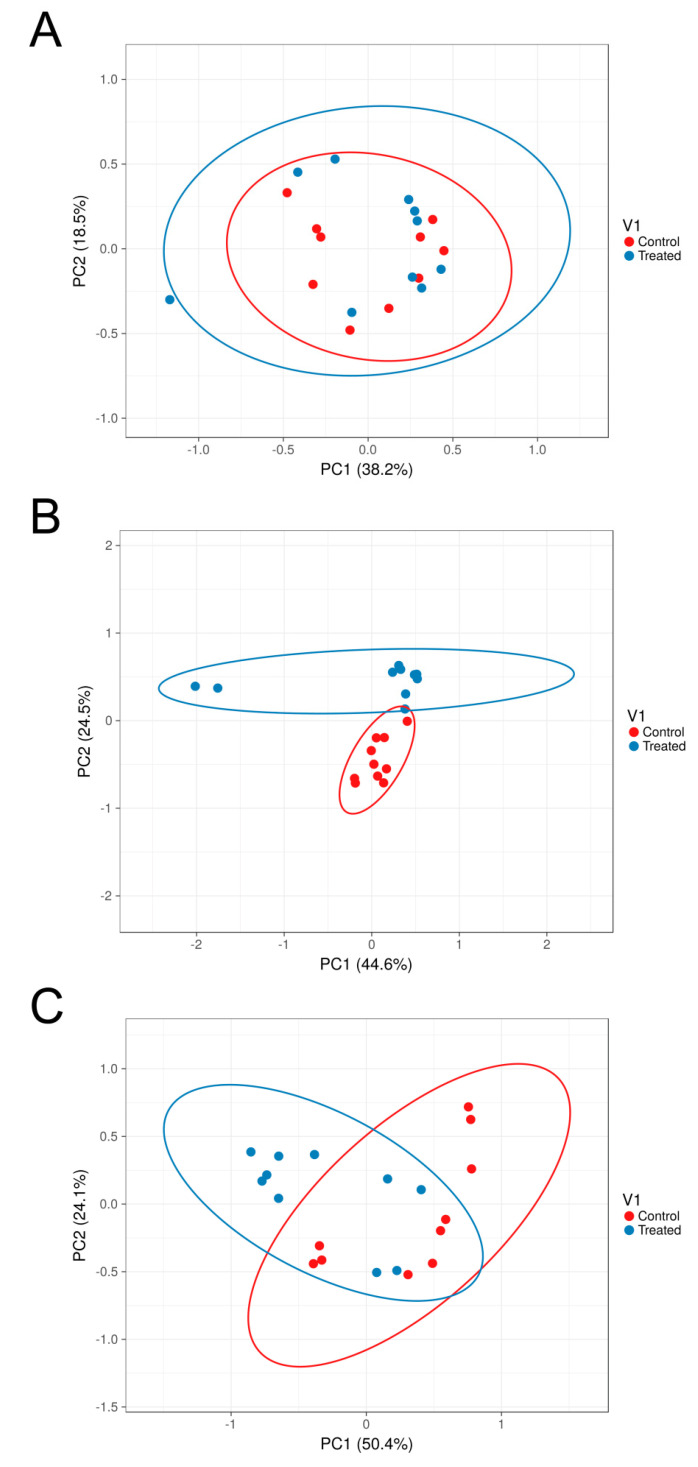
Beta diversity Principal Coordinates Analysis between traditionally (*n* = 10) and alternatively fed water buffaloes (*n* = 10). PCoA analysis based on Weighted UniFrac distance matrices between traditionally and alternatively fed water buffaloes in the rumen (*p*-value = 0.25) (**A**), large intestine (*p*-value = 0.002) (**B**) and feces (*p*-value = 0.009) (**C**).

Beta diversity analysis was carried out by comparing distances between groups (traditional vs. alternative diet) for each body site (rumen, large intestine and feces) by both taxonomic and phylogenetic approaches. Results displayed significant differences in all the performed comparisons with the exception of the weighted UniFrac metric for rumen samples (Table 4) and indicated that for each body site, the structure of the microbiota exhibited significant differences in both species abundance and presence between the two different diets. The weighted UniFrac distance matrices were used for the PCOA analysis to evaluate the variation of microbiota structure between traditional and alternative feeding for each body site (Figure 6). In this analysis, each sample is represented by a single point, and the closer two points are the more similar the microbiota of those samples. Results showed that, while rumen samples from both diet-based groups clustered together, distinct grouping could be observed in the large intestine and feces based on diet, with samples clustering mostly along PC1, which could explain 38.2%, 44.6% and 50.4% of the variations in the rumen, large intestine and feces, respectively.

The Permutational Analyses of Multivariate Dispersions (PERMDISP) indicated that there was no heterogeneity in multivariate dispersion within groups, either in the rumen (PERMDISP—Bray–Curtis: *F*-value = 0.190, *p*-value = 0.683; Unweighted UniFrac: *F*-value = 1.403, *p*-value = 0.285; Weighted UniFrac: *F*-value = 0.987, *p*-value = 0.354), or intestine (PERMDISP—Bray–Curtis: *F*-value = 2.590, *p*-value = 0.054; Unweighted UniFrac: *F*-value = 1.509, *p*-value = 0.075; Weighted UniFrac: *F*-value = 0.959, *p*-value = 0.411) or feces (PERMDISP—Bray–Curtis: *F*-value = 3.485, *p*-value = 0.075; Unweighted UniFrac: *F*-value = 4.231, *p*-value = 0.092; Weighted UniFrac: *F*-value = 0.153, *p*-value = 0.688).

**Figure 7:**
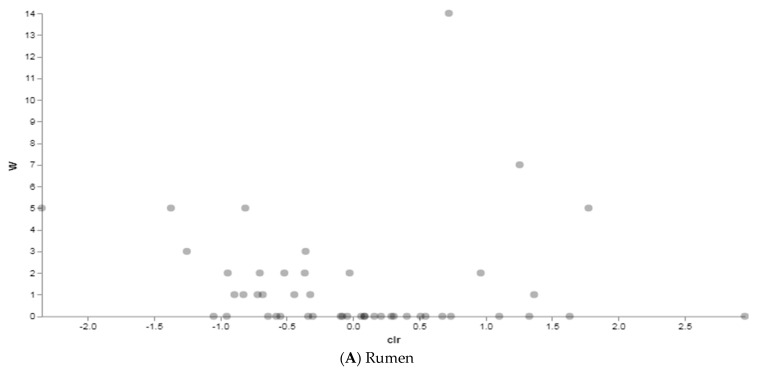

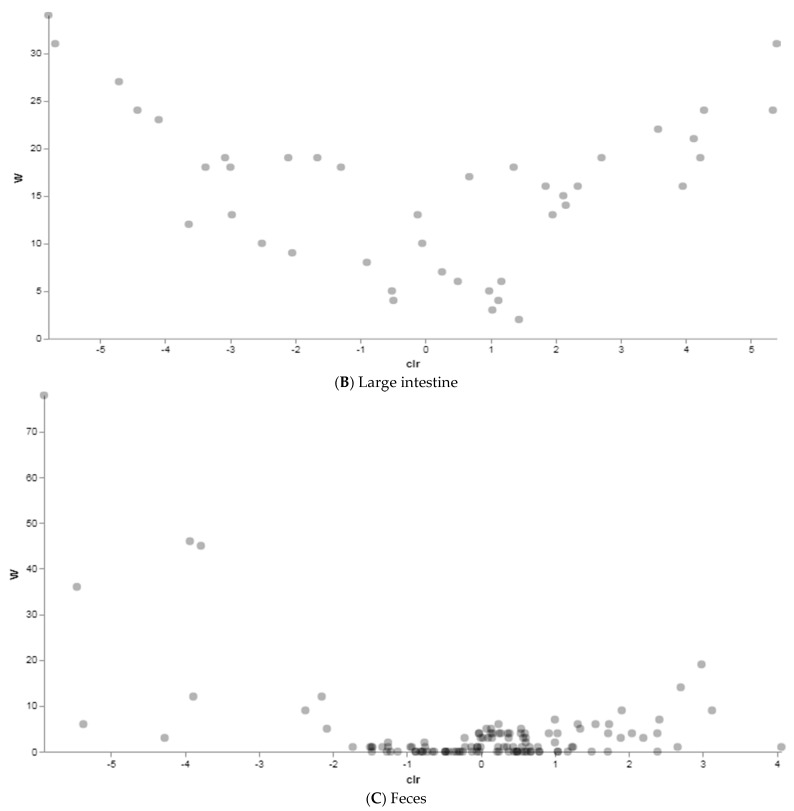
ANCOM results between traditionally (*n* = 10) and alternatively (*n* = 10) fed water buffaloes. Dots represent features identified by ANCOM as differentially abundant between groups. Statistically significant differentially abundant taxa are represented by the dots in the upper corners of the graph.

Then, we investigated how the different diets influenced the relative abundance of microbial features at each body site by the Analysis of Composition of Microbiomes (ANCOM) in order to identify features that significantly differed in abundance between the two groups (traditionally vs. alternatively fed water buffaloes) in the rumen, large intestine and feces, respectively. Between rumen content samples, a total of eight taxa were identified as differentially abundant in differentially fed animals, even though with low W values (Figure 7A and Suppl. Table S3). Three taxa appeared more abundant in the rumen of traditionally fed animals: an uncultured family of the Order Rhodospirillales (W = 14, clr F-statistic = 0.7), an uncultured Order of the Class Bacilli (W = 7, clr F-statistic = 1.26), and the family Endomicrobiaceae (W = 5, clr F-statistic = 1.8). Four taxa resulted more abundant in the alternatively fed water buffaloes: Families RF39 (W = 5, clr F-statistic = −1.4), Prevotellaceae (W = 5, clr F-statistic = −0.8), Succinivibrionaceae (W = 3, clr F-statistic = −1.3), and F082 (W = 3, clr F-statistic = −0.4). In the large intestine, a total of seven genera differentially abundant between groups were identified, with *Paeniclostridium* (W = 31, clr F-statistic = 5.4), *Saccharofermentans* (W = 24, clr F-statistic = 4.3) and *Prevotella* (W = 24, clr F-statistic = 5.3) more abundant in traditionally fed water buffaloes, and *p*-2534-18B5_gut_group (W = 34, clr F-statistic = −5.8), *Alistipes* (W = 31, clr F-statistic = −5.7), the Uncultured Genus 004 of the Prevotellaceae family (Prevotellaceae_UCG-004) (W = 27, clr F-statistic = −4.7) and *Bacteroides* (W = 24, clr F-statistic = −4.4) more abundant in alternatively fed animals (Figure 7B and Suppl. Table S3). Finally, in feces (Figure 7C and Suppl. Table S3), the genus *Solibacillus* was found to be significantly more abundant in alternatively fed animals (W = 78, clr F-statistic = −5.9). These data are consistent with the beta diversity analysis results indicating more significant differences in microbiota structure in the large intestine and feces, rather than in rumen content.

### 3.4. Diet Digestibility

Lipid (FC), energy (GE) and fiber (NDF and ADF) digestibility were higher (*p* < 0.01) in traditionally fed buffaloes compared to alternatively fed buffaloes. On the contrary, ash digestibility was higher (*p* < 0.01) in alternatively fed animals. No differences were found for crude protein (CP) and organic matter (OM) (Table 5).

## 4. Discussion

The microbial community situated in the Gastrointestinal tract (GIT) is essential for the health of ruminant livestock and affects the productions of farmed animals. Manipulation of the gut microbiota through diet has been proposed as a tool for improving animal health and productions such as milk. Therefore, the characterization of the GIT microbiota under different fiber contents and energy/protein ratios in the diet can contribute to understanding the relationships between the host and its microbiota, with possible influence on the health status and production efficiency of the animals. In this study, we describe the microbiota of the rumen, large intestine and feces of water buffaloes fed two different diets, characterized by different NSC/CP ratios. Our results indicated that there are significant differences between the considered body sites within the same group of animals, for both groups. Moreover, for each tract, the comparison between the microbial compositions observed for different feeding systems indicated that diet does not influence bacterial richness or evenness, but can significantly shape the structure of microbiota by inducing differences in both species abundance and presence. This result was particularly evident in the large intestine, where beta diversity analysis exhibited the largest difference between the two diet-based groups.

The taxonomic analysis of the microbial populations characterized in the different GIT sites indicated that the most abundant phyla were Bacteroidetes, Firmicutes and Proteobacteria in variable order depending on the gut site. The Phylum Bacteroidetes, mostly represented by the families Prevotellaceae and Rikenellaceae, resulted to be more abundant in the rumen, while the Phylum Firmicutes was more abundant in the large intestine and feces. In the large intestine, Firmicutes were mostly represented by the families Peptostreptococcaceae, Erysipelotrichaceae, Christensenellaceae, Clostridiaceae, Lachnospiraceae and Oscillospiraceae, while in feces this Phylum mostly included Lachnospiraceae, Oscillospiraceae, *UCG-010*, Christensenellaceae and the Coprostanoligenes_group. The predominance of Bacteroidetes and Firmicutes observed in this study is consistent with previous data showing that these phyla account for over 80% of the microbial community of the gastrointestinal tract of water buffalo and herbivorous mammals in general [1,13,42], with Firmicutes being the most represented Phylum in the core microbiota of milk from healthy water buffaloes [43]. Indeed, the phylum Bacteroidetes includes several bacterial genera able to produce enzymes that can degrade oligosaccharides, polysaccharides such as celluloses, pectins and xylans, as well as host-derived carbohydrates such as mucins and N-glycans [24,42,43,44,45,46]. Similarly, the Phylum Firmicutes plays an important role in fiber digestion as it includes several fiber-adherent bacterial species [28]. These two phyla include most of the Gram-negative bacteria such as the genera *Eubacterium* and *Succinivibrio*, which are able, in the GIT, and particularly in the rumen, to utilize acetate to produce butyrate, which is an important energy source for both the host and the gut microbiota. Moreover, species belonging to the genera *Clostridium*, *Butyrivibrio* and *Prevotella* can produce hydrolytic enzymes such as ferulic acid esterases (FAE) which can contribute to plant cell wall degradation thus improving the access of main chain degrading enzymes and digestibility of high fiber diets [1]. The genus *Ruminococcus* includes cellulolytic species able to produce acetate, formate and succinate from cellulose [4]. Regarding the rumen, which harbors the largest portion of the bovine GIT by volume and is the most well-characterized GIT site for the residing microbial community, our findings highlighted the presence of a core microbiota including, among its members, *Prevotella*, *Bacteroidales* UCG-001, Christensenellaceae, Rikenellaceae RC9_gut_group, Ruminococcaceae NK4A214_group, Oscillospiraceae, *Ruminococcus* and *Eubacterium*. These taxa have been found to belong to the core microbiota of the rumen in bovine [3,47,48] as well as in other mammals, including sheep [49], horses [50] and pigs [51]. Moreover, in our study, these same taxa were found in the core microbiota of the large intestine and feces too, therefore resulting as fundamental members of the GIT core microbiota of water buffalo. These taxa are indeed ubiquitous throughout the bovine gut and in other herbivorous mammals, as they are involved in fiber digestion [1,52]. However, the rumen, intestine and fecal microbiota appeared clearly separated according to their microbial composition. Indeed (among the most represented genera), the genera *Ruminobacter*, *Butyrivibrio*, *Succiniclasticum*, *Fibrobacter*, *Succinivibrionaceae* UCG-002, *Papillibacter*, *Bacteroidales* BS11 gut group, *Succinivibrio*, *Saccharofermentans*, and *Lachnospiraceae* UCG-008 and NK3A20 group were among those identified as strongly linked with the rumen microbiota. These genera are all involved in carbohydrates fermentation leading to the production of volatile fatty acids, CO_2_ and H_2_ [52]. Some of these genera are able to degrade only specific substrates, while some other genera, such as *Butyrivibrio* and *Fibrobacter*, can metabolize both structural and nonstructural carbohydrates [52]. The genera *Bacteroides*, *Alistipes*, *Rikenellaceae*, *Turicibacter*, *Clostridium* (*sensustricto*), and *Escherichia*-*Shigella* resulted as members of the core microbiota of both the large intestine and feces, even though these sites exhibited the presence of peculiar members of their own core microbiota. The association of *Bacteroides* and *Alistipes* with feces rather than the rumen can be explained by the ability of the former to degrade both dietary polysaccharides from plant cell walls that the host’s enzymes cannot and host mucins [53] and the inability of the latter to degrade polysaccharides [54], which are indeed less abundant in feces than in the rumen. The family Rikenellaceae includes members able to produce, through fermentation pathways, propionate, which is the principal gluconeogenic substrate in ruminants [55], and acetate, which is one of the major substrates for de novo lipogenesis [56]. Finally, the genus *Clostridium*, particularly the one classified as *sensustricto* (cluster I), produces butyrate, a short-chain fatty acid, which is a primary energy source for the development of GIT epithelial cells [57].

The administration of an alternative diet with a higher NSC/CP ratio, induced several differences in the microbiota of the investigated gut sites, mostly characterized by differences in the overall community structures (beta diversity indexes) with comparable taxa abundance and evenness (alpha diversity indexes). These results are consistent with the differences observed in calves raised on low or high-fiber diets [58] and hence with high or low NSC amounts. The traditional diet was characterized by a lower NSC/CP ratio compared to the alternative one, that increased lipid and fiber digestibility and improved energy availability. Our findings showed that alternatively fed animals displayed an increase of Fibrobacteres and Proteobacteria, with a decrease of Firmicutes and Verrucomicrobiota in all the analyzed sites. Firmicutes improve feed conversion ratio [11,59]. In this trial, better digestibility in the traditional compared to alternative diet was shown probably due to a higher Firmicutes population [60]. On the other hand, in alternatively fed animals we found a lower F/B ratio, particularly in the large intestine. This effect might be determined by the presence of lycopene in tomato peel. Indeed, many studies have proposed that polyphenols such as lycopene can exert prebiotic effects by promoting beneficial bacteria growth and controlling potential pathogenic bacteria, thus preserving the structure of gut microbiota and contributing to the maintenance of host health [61]. Consistently, in ruminants, phenolic compounds contained in the cashew nut shell liquid have been reported to successfully direct rumen fermentation toward higher propionate production and lower ammonia and methane emissions, without affecting VFAs production or diet digestibility [62]. F/B ratio is considered an important parameter to define the health status both in humans and animals [61] and has been shown to be affected by the presence of lycopene in the diet [63]. It has been proposed that polyphenols can repress Firmicutes, thus favoring Bacteroidetes in the gut [64]. Other possible effects on animal host heath might have been exerted by the alternative diet administered in this study [65], however, due to the short experimental duration, we can speculate that no discernable difference could be attributed to the tomato peel alone.

Ruminants, similarly to monogastric animals, receive nutritive benefits from the feed fermentation at the colon level [66]. In our study, differences were found for a total of seven genera. In particular, a high level of *Saccharofermentans*, which produces acetate at the gut level, and *Prevotella*, which improves feed efficiency, were found in traditionally fed animals compared with their alternative counterparts [67]. Moreover, in both the large intestine and feces, Bacteroidetes and Fibrobacteres resulted to be more abundant in alternatively fed water buffaloes compared with the traditionally fed ones. Beta analysis displayed a lower variation in the rumen microbiota if compared to that of intestine and feces, in response to different NSC/CP ratios contained in the diet. These results can be explained by the more stringent ecological characteristics of the rumen, which acts as an anaerobic fermenter chamber, with a pH between 5.5 and 6.9, and a temperature between 38–40 °C [1]. Our findings indicated a number of differentially abundant taxa between traditional and alternative fed water buffaloes. The alternative diet induced a significant increase, among the others, of the families Prevotellaceae and Succinivibrionaceae, with the genera *Alistipes*, Prevotellaceae_Uncultured Genus-004 and *Bacteroides* found to be more abundant in the large intestine. Interestingly, in this body site, the two different diets induced differential abundances of closely related genera, that is *Prevotella* in traditionally fed water buffaloes, and Prevotellaceae_Uncultured Genus-004 in the alternatively fed ones. Further studies will be necessary on these genera to reveal why the diet encouraged one and not the other.

Overall, these results support the possibility to manipulate the composition of gut microbiota in ruminants as a useful tool for the management of dairy farms. Indeed, many of the most represented genera in the GIT have been correlated to both the health status of the host and its milking performance, through a symbiotic interaction that provides nutritional, developmental, physiologic and immunological benefits to the host. Commensal bacteria can modulate both innate and adaptive immune response, are related to barrier enhancement and can stimulate the establishment of a healthy microbiota, inhibiting at the same time the colonization by pathogens [2]. The reconstitution of healthy microbiota is an effective method for preventing or treating gastrointestinal disorders, and fecal microbiota transplantation (FMT) has been successfully applied to ameliorate diarrhea in growing calves. In a recent study on FMT in calves, Kim and colleagues [68] found that the families Christensenellaceae, Clostridiaceae, Peptostreptococcaceae, Dehalobacteriaceae and Coriobacteriaceae were more abundant in FMT cattle, thus suggesting their possible association with growth and/or fattening in growing cattle. As specifically regards milking performance in water buffalo, *Butyrivibrio* has been shown to be positively correlated with average milk fat yield, average milk total solid yield and standard milk yield, while *Acinetobacter* has been positively correlated with average protein milk yield, average milk total solid yield and standard milk yield [13]. Finally, the genus *Prevotella* has been shown to influence milk performance in both water buffalo and cattle [11,12,13,69].

A limitation of this study is the relatively short experimental period. Indeed, further research will be necessary to demonstrate the stability of the observed changes in microbiota composition in order to exclude the transient effects of the diet on microbial species presence and their abundance and to determine that these results could also be obtained in different environmental conditions. Furthermore, a follow-up study would be useful to provide data on eventual differences in production performance and/or metabolic activity of differentially fed animals.

## 5. Conclusions

In conclusion, our results indicate a difference in diet digestibility linked to microbiota modification at the GIT level conditioned by energy/protein levels (NSC/CP) during the dry period in water buffalo species. Overall, this study characterized the microbial communities of the gastrointestinal tract of water buffalo and evaluated the influence of a different energy/protein (NSC/CP) ratio in the diet on the GIT microbiota structure. Our results indicated that Bacteroidetes, Firmicutes and Proteobacteria were the most abundant phyla in all the GIT sites. Alternatively fed animals displayed an increase of Fibrobacteres and Proteobacteria, with a decrease of Firmicutes and Verrucomicrobiota in all the analyzed sites. The obtained results indicate that diet can strongly influence the microbiota of different GIT sites and might therefore act as a modulating factor for specific GIT microorganisms able to affect the productive performance of adult animals. 

## Figures and Tables

**Table 1:** Feed and chemical composition of buffalo diets characterized by different NSC/CP ratios in traditionally and alternatively fed buffaloes.

Feed	Diet (kg)	
	**Traditional**	**Alternative**
Triticale Silage	8.0	
Tomato peel		12.0
Wheat Straw	6.0	7.0
Concentrate	2.0	2.2
Total	16	21.2
	**Composition (% Dry Matter Intake)**	
Dry Matter (kg)	9.7	9.4
CP	6.9	6.0
Fat	2.8	2.1
NDF	65.8	68.1
NSC	13.2	13.8
Ash	11.3	10.0
NSC/CP	1.9	2.3

CP: Crude Protein; NDF: Neutral Detergent Fiber; NSC: Non-Structural Carbohydrates.

**Table 2:** Most abundant Families and Genera found in the analyzed gastrointestinal sites. Relative abundance (mean frequency ± SD) of the most abundant Families and Genera observed in the analyzed GIT sites.

Genera					
**Mean**	**SD**	**Rumen Traditional ^a^**	**Mean**	**SD**	**Rumen Alternative**
19.0	5.4	g_Prevotella	25.3	1.7	g_Prevotella
12.3	3.2	g_Rikenellaceae_RC9_gut_group	7.4	1.7	g_Rikenellaceae_RC9_gut_group
6.4	3.7	g_Christensenellaceae_R-7_group	6.7	2.6	g_Christensenellaceae_R-7_group
4.3	2.9	g_Ruminobacter	6.5	3.7	g_Ruminobacter
4.2	1.4	g_Succiniclasticum	3.4	0.9	g_Succiniclasticum
4.1	1.8	g_Butyrivibrio	3.3	4.1	g_Succinivibrionaceae_UCG-002
3.1	0.9	g_F082	3.1	1.9	g_NK4A214_group
3.1	0.8	g_Papillibacter	3.0	6.2	g_Acinetobacter
2.8	1.3	o_Rhodospirillales;f__uncultured;g__uncultured	2.9	1.0	g_Butyrivibrio
2.6	4.4	g_Succinivibrionaceae_UCG-002	2.6	0.5	g_F082
2.2	0.6	g_Lachnospiraceae_AC2044_group	2.5	0.7	g_Candidatus_Saccharimonas
2.2	0.7	g_NK4A214_group	2.2	1.0	g_Ruminococcus
2.2	1.6	g_Fibrobacter			
2.1	4.6	g_Escherichia-Shigella			
2.1	0.7	g_Candidatus_Saccharimonas			
**Mean**	**SD**	**Intestine Traditional**	**Mean**	**SD**	**Intestine Alternative**
19.9	7.4	f_Peptostreptococcaceae;	17.8	12.7	g_Escherichia-Shigella
15.5	18.5	g_Escherichia-Shigella	9.4	24.0	g_Aeromonas
9.8	5.9	g_Turicibacter	8.3	24.2	g_Shewanella
8.9	4.7	g_Christensenellaceae_R-7_group	8.1	4.7	g_Rikenellaceae_RC9_gut_group
7.9	4.2	g_Romboutsia	3.8	3.8	g_Solibacillus
5.6	3.2	g_Paraclostridium	3.6	3.4	g_Bacteroides
5.1	3.2	g_Clostridium_sensu_stricto_1	3.5	6.0	g_Lysinibacillus
3.4	4.0	g_Prevotella	3.4	2.0	g_UCG-005
2.7	2.5	g_Paeniclostridium	2.8	5.8	g_Acinetobacter
2.4	1.9	g_Candidatus_Saccharimonas	2.5	1.9	f_Lachnospiraceae;
2.1	2.6	g_Rikenellaceae_RC9_gut_group			
**Mean**	**SD**	**Feces Traditional**	**Mean**	**SD**	**Feces Alternative**
9.9	4.0	g_Rikenellaceae_RC9_gut_group	13.5	14.5	g_Acinetobacter
6.9	2.5	g_Bacteroides	11.7	8.9	g_Escherichia-Shigella
5.0	1.8	f_Lachnospiraceae;	8.9	1.7	g_Rikenellaceae_RC9_gut_group
4.5	1.5	g_Alistipes	4.3	2.0	g_Bacteroides
4.4	6.6	g_Escherichia-Shigella	4.2	2.3	g_Bacteroidales_RF16_group
4.2	2.2	g_Bacteroidales_RF16_group	3.8	1.1	f_Lachnospiraceae;
4.1	0.8	f_Oscillospiraceae;g_uncultured	3.5	2.8	g_Solibacillus
3.9	1.5	g_UCG-010	3.5	1.7	g_UCG-005
3.8	1.2	g_UCG-005	3.1	1.0	g_Alistipes
3.7	2.4	g_Christensenellaceae_R-7_group	2.8	2.1	g_UCG-010
3.2	1.0	g_Eubacterium_coprostanoligenes_group	2.8	1.1	g_Eubacterium_coprostanoligenes_group
2.8	0.9	o_Bacteroidales;f_uncultured;g__uncultured	2.4	2.3	g_Lysinibacillus
2.7	1.6	f_Lachnospiraceae;g_uncultured			
2.5	2.3	g_Alloprevotella			
2.3	0.9	g__Prevotellaceae_UCG-004			
2.1	1.4	c__Gammaproteobacteria;			

^a^ In the taxa list, the letter g_ indicates Genus, f_ indicates Family, c_ indicates Class, o_ indicates Order.

**Table 3:** Alpha diversity analysis of GIT microbiota characterized in traditional vs. alternatively fed animals.

Body Site	Test	Index/Matrix	H	*p*-Value
**Rumen**	Kruscal–Wallis	Observed ASVs	0.516102	0.472509
	Kruscal–Wallis	Pielou evenness	2.765714	0.096304
**Large intestine**	Kruscal–Wallis	Observed ASVs	1.651429	0.198765
	Kruscal–Wallis	Pielou evenness	1.285714	0.256839
**Feces**	Kruscal–Wallis	Observed ASVs	0.012867	0.909688
	Kruscal–Wallis	Pielou evenness	0.012867	0.909688

**Table 4:** Beta diversity analysis of GIT microbiota characterized in traditional vs. alternatively fed animals.

Body Site	Test	Matrix	Pseudo-F	*p*-Value
**Rumen**	PERMANOVA	Bray–Curtis	3.007807	0.001
	PERMANOVA	UnWeighted UniFrac	2.301941	0.007
	PERMANOVA	Weighted UniFrac	1.400113	0.25
**Large intestine**	PERMANOVA	Bray–Curtis	10.595599	0.001
	PERMANOVA	UnWeighted UniFrac	10.118457	0.001
	PERMANOVA	Weighted UniFrac	4.569653	0.002
**Feces**	PERMANOVA	Bray–Curtis	3.609716	0.002
	PERMANOVA	UnWeighted UniFrac	3.074413	0.009
	PERMANOVA	Weighted UniFrac	5.124755	0.009

**Table 5:** Digestibility of buffalo diets characterized by different NSC/CP ratios in traditionally fed and alternatively fed buffaloes.

Constituent of Diet	Traditionally Fed	Alternatively Fed
OM	74.3 ± 0.7	76.6 ± 0.7
CP	69.0 ± 1.4	65.1 ± 1.4
FC	85.9 ± 0.9 ^A^	80.5 ± 0.9 ^B^
NDF	81.4 ± 0.5 ^A^	76.9 ± 0.5 ^B^
ADF	64.0 ± 0.5 ^A^	59.2 ± 0.1 ^B^
Ash	42.3 ± 1.3 ^A^	54.2 ± 1.3 ^B^
GE	82.9 ± 0.3 ^A^	77.0 ± 0.4 ^B^

OM, organic matter (=100-ash); CP, crude protein; FC, fat content; NDF, neutral detergent fiber; ADF, acid detergent fiber, GE (Gross energy). Results are means ± SEM. ^A,B^, *p* < 0.01.

## Data Availability

Data available in a publicly accessible repository. The data presented in this study are openly available in NCBI under BioProject accession number PRJNA675438.

## Supplementary Materials

The following are available online at https://www.mdpi.com/article/10.3390/vetsci8060096/s1; Table S1: Features present in the negative controls included in the study. Feature ID, Frequency, Frequency in study samples, Number of samples and Taxonomic classifications of the features found in the negative controls included in the present study. Table S2:Core microbiota at Family and Genus level in traditionally and alternatively fed water buffaloes. List of the Families and Genera shared by at least 80% of samples for each GI site. Table S3: Features differentially abundant in rumen, large intestine and feces between differentially fed water buffaloes (ANCOM results). Figure S1. Core microbiota at Family level of the rumen, large intestine and feces from differentially fed water buffaloes. Families shared by at least 80% of samples included in each body site: (**A**) traditionally fed water buffaloes; (**B**) alternatively fed water buffaloes.
