# Supplementary material for: Different Non-Structural Carbohydrates/Crude Proteins (NCS/CP) Ratios in Diet Shape the Gastrointestinal Microbiota of Water Buffalo
Source: Vet Sci. 2021 May 31;8(6):96. doi: 10.3390/vetsci8060096 (PMC8229247; doi:10.3390/vetsci8060096)

Supplementary Figure S1_Family core. Core microbiota at Family level of rumen, large intestine and feces from differentially fed water buffaloes. Families shared by at least 80% of samples included in each body site: (A) traditionally fed water buffaloes; (B) alternatively fed water buffaloes.

A Families included in the core microbiota in traditionally fed water buffaloes.

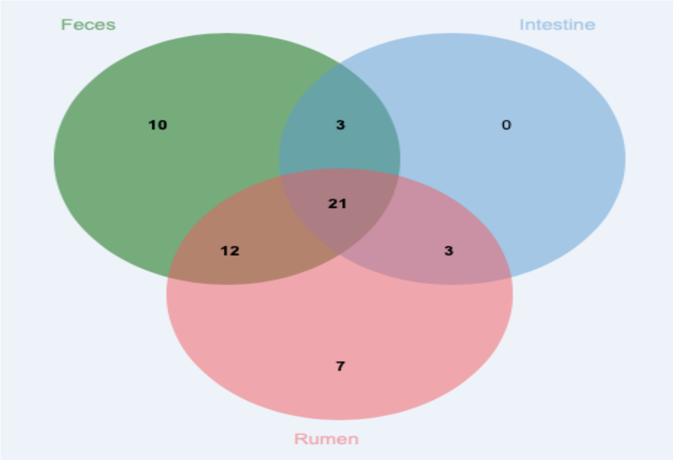

B Families included in the core microbiota in alternatively fed water buffaloes.

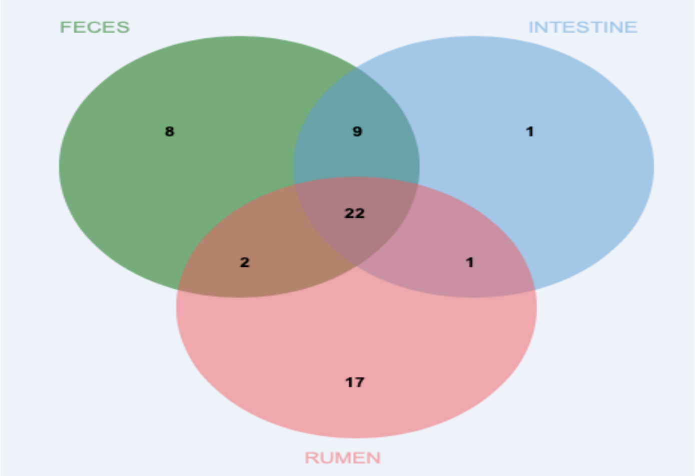

Supplement: Supplementary file 1 [file vetsci-08-00096-s001.zip › Suppl_Fig 1_Family Core.pdf]
